# YOLO-AR: An Improved Artificial Reef Segmentation Algorithm Based on YOLOv11

**DOI:** 10.3390/s25175426

**Published:** 2025-09-02

**Authors:** Yuxiang Wu, Tingchen Jiang, Zhi Xi, Fei Yin, Xiuping Wang

**Affiliations:** 1 College of Marine Technology and Surveying, Jiangsu Ocean University, Lianyungang 222005, China; 2023210202@jou.edu.cn (Y.W.); wxp313@sina.com (X.W.); 2 Lianyungang Water Resources Bureau, Lianyungang 222061, China; xz59138333@163.com (Z.X.); lygshzzbgs@163.com (F.Y.)

**Keywords:** artificial reef detection, deep learning, multibeam sonar images, YOLOv11

## Abstract

Artificial reefs serve as a crucial measure for preventing habitat degradation, enhancing primary productivity in marine areas, and restoring and increasing fishery resources, making them an essential component of marine ranching development. Accurate identification and detection of artificial reefs are vital for ecological conservation and fishery resource management. To achieve precise segmentation of artificial reefs in multibeam sonar images, this study proposes an improved YOLOv11-based model, YOLO-AR. Specifically, the DCCA (Dynamic Convolution Coordinate Attention) module is introduced into the backbone network to reduce the model’s sensitivity to complex seafloor environments. Additionally, a small-object detection layer is added to the neck network, along with the ultra-lightweight dynamic upsampling operator DySample (Dynamic Sampling), which enhances the model’s ability to segment small artificial reefs. Furthermore, some standard convolution layers in the backbone are replaced with ADown (Advanced Downsampling) to reduce the model’s complexity. Experimental results demonstrate that YOLO-AR achieves an mAP@0.5 of 0.912, an intersection-over-union (IOU) of 0.832, and an F1 score of 0.908. Meanwhile, the parameters and model size of YOLO-AR are 2.67 million and 5.58 MB. Compared to other advanced segmentation models, YOLO-AR maintains a more lightweight structure while delivering a superior segmentation performance. In real-world multibeam sonar images, YOLO-AR can accurately segment artificial reefs, making it highly effective for practical applications.

## 1. Introduction

Marine ecological security is an important foundation for the development of marine business [1]. In recent years, the development and use of global marine resources have intensified. This has caused more and more threats to the health of marine ecosystems. To deal with this, people have taken a lot of measures. These measures aim to reduce the bad effects of human activities and restore the damaged marine habitats [2]. Among them, marine ranches play a significant role in improving the marine ecological environment and promoting sustainable ecological development [3,4]. Artificial reefs are an artificially constructed underwater ecosystem. They have been widely used in many countries and regions. Building and setting up artificial reefs are important technological methods in marine ranching [5]. The surface and internal environment of artificial reefs provide suitable habitats and hiding places for marine organisms [6,7]. So, they play an important part in increasing biodiversity and helping fishery development [8,9,10]. Their three-dimensional structure creates more hiding places and feeding opportunities. It also influences species interactions, such as settlement, competition, and hunting behavior. This helps to improve the underwater environment and the variety of aquatic life. It also helps with the long-term sustainable use of fishery resources [11,12]. Numerous studies have shown that artificial reefs generally help improve fishery resources in nearby areas. This improvement is linked to increased productivity and the attraction and aggregation of fish in the region [13,14]. However, artificial reefs are susceptible to burial by sediment due to water flow erosion, necessitating regular monitoring. Accurately detecting and assessing artificial reefs is of great significance for evaluating marine ranching biological resources [15]. Traditional methods for monitoring artificial reefs, such as diver-based surveys and underwater cameras, are inefficient, costly, and unsuitable for large-scale reef monitoring [16]. Sonar uses acoustic wave technology for target detection and can penetrate dark and murky waters, making it a key detection method in underwater environments [17,18]. A multi-beam echo sounder can perform high-precision depth measurement and provide backscatter images of the seabed [19]. It is currently widely used in seabed measurement [20,21]. While multibeam sonar can efficiently detect artificial reefs on the seafloor, the detection of artificial reefs in sonar images still largely relies on manual identification. Manual detection accuracy depends on individual expertise and image quality, leading to low precision and efficiency. To tackle these challenges, deep learning-based object detection has increasingly attracted research attention. One of the central concerns in current studies is how to refine existing models and develop deep learning frameworks that are better tailored to the specific demands of artificial reef detection.

## 2. Related Work

As marine resources keep developing, underwater target detection has become a key point in both marine engineering and ecological monitoring. Traditional methods for underwater image processing usually depend on manual feature extraction. When this is combined with the complexity of underwater environments, it leads to limited detection accuracy and a poor generalization performance. On the other hand, the appearance of deep learning has brought a hopeful alternative. It has strong abilities in automatic feature learning, and these abilities can help solve those technical problems [22]. In the past few years, object detection based on deep learning has been used in many different fields. This has led to a lot of studies that aim to improve underwater target detection. Zhang et al. (2025) [23] proposed a novel detection framework, UUVDNet, which incorporates an enhanced training strategy and attention mechanisms for sonar image detection tasks, improving both detection accuracy and speed. Šiaulys et al. (2024) [24] applied deep learning models to perform segmentation on underwater images of reefs in the southeastern Baltic Sea, estimating the coverage of benthic habitats. Their findings confirm that deep learning models can achieve the required accuracy for underwater sonar image detection while significantly outperforming manual annotation in efficiency. Marre et al. (2020) [25] designed a custom network that classifies coral reefs with high accuracy. Using this model, they assessed reef biodiversity and ecological conditions through the Coral Assemblage Index (CAI) and Shannon Index, providing a cost-effective and efficient tool for coral reef analysis. Li and Zhang (2024) [26] proposed a lightweight underwater garbage segmentation network, effectively enabling fine-grained localization and recognition of debris in complex underwater environments, thus contributing to improved marine waste recycling rates. Li et al. (2023) [27] developed the MA-YOLOv7 network to detect objects in sidescan sonar images. This algorithm achieved a state-of-the-art performance and is suitable for real-world underwater applications. Shi et al. (2024) [28] proposed an advanced detection framework using EfficientNet as the backbone network, combining efficient feature extraction with multi-scale feature fusion. Their framework demonstrated high accuracy in sonar image target detection and effectively improved the performance under noisy conditions. Qin et al. (2024) [29] introduced a model called YOLOv7C, which achieved a 1.9% increase in average precision.

The integration of object detection and segmentation has become a major trend in computer vision. As a representative of single-stage detection frameworks, the YOLO series has demonstrated unique value in underwater scenarios requiring real-time processing, thanks to its efficient detection speed and end-to-end architecture. From YOLOv5 onward, segmentation heads were introduced, marking the framework’s formal entry into the field of instance segmentation. YOLO has long been a research hotspot in object detection and has now shown an excellent performance in segmentation as well. Yang et al. (2025) [30] utilized emerging Large Vision Models (LVMs) along with the YOLOv5 model to propose a simple yet powerful teacher–student framework (TeSF). This framework achieved a good balance between segmentation accuracy and computational efficiency on the Shanghai Metro tunnel surface defect dataset. Lin et al. (2025) [31] proposed a novel deep learning framework, Multi-Scale Task-Aligned YOLO (MSTA-YOLO), which effectively segments retinal ganglion cells labeled with different markers. Silpalatha et al. (2025) [32] leveraged the latest innovations in the YOLO architecture to optimize the speed and accuracy of remote sensing image segmentation tasks. Their model accurately handled geometric complexities in image data, achieving improvements in accuracy, precision, recall, and IOU while significantly reducing the processing time compared to traditional segmentation methods. Shams et al. (2025) [33] used YOLOv8 for broiler segmentation, significantly improving the weight estimation accuracy without requiring size measurements, making the process more efficient and convenient. Shen et al. (2025) [34] proposed an efficient instance segmentation model, MSA-YOLO, based on YOLOv8, which greatly enhanced the accuracy and robustness of grape peduncle instance segmentation. Su et al. (2022) [35] introduced a dual-lens model that simultaneously utilizes YOLO and LOGO architectures for quality inspection and segmentation. Their model demonstrated higher efficiency and a superior performance compared to previous approaches, reducing computational demands and improving the generalization and accuracy of computer-aided breast cancer diagnosis. Xu et al. (2025) [36] enhanced the lightweight segmentation network BiSeNet and integrated it into the YOLOv5 network. Their approach achieved a better balance between accuracy and efficiency in real-world coal mining applications.

Currently, deep learning-based object detection research has been applied across various domains, and its use in underwater target detection and recognition is attracting increasing attention. However, research on artificial reef group detection remains limited, particularly in the field of artificial reef segmentation models. At present, artificial reef segmentation still faces several challenges. In sonar images, artificial reefs often appear as small targets and may be partially buried by sediment, leading to severe missed detections of small objects. Moreover, sonar images are characterized by strong noise and low contrast, with minimal grayscale differences between artificial reefs and their surroundings, which can easily result in false detections.

To address these issues and achieve accurate, efficient detection of artificial reefs in sonar images—facilitating effective reef monitoring—this study proposes a YOLOv11-based detection framework capable of high-precision segmentation of artificial reefs in multibeam sonar imagery. First, based on the Coordinate Attention (CA) mechanism, a spatial attention module tailored for artificial reef semantic segmentation, termed DCCA, is introduced. The DCCA module is integrated into the backbone network to enhance feature extraction capability. Subsequently, an additional small-object detection layer and an extra detection head are added in the neck. Furthermore, an ultra-lightweight dynamic upsampling operator, DySample, is incorporated into the neck to refine the upsampling process, improving model performance while minimizing computational resource consumption. To further achieve network lightweighting, conventional convolution layers in the backbone are replaced with the ADown downsampling module, reducing model complexity.

## 3. Materials and Methods

### 3.1. Image Annotation and Dataset Construction

**Figure 1:**
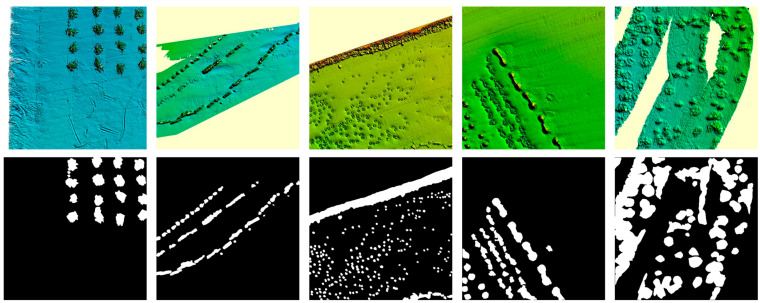
Selected artificial reef images and annotations.

Deep learning-based segmentation requires a large amount of data for training to achieve a high recognition accuracy. In this study, we utilize the FIO-AR dataset, a multibeam sonar image artificial reef detection dataset created by Dong et al. (2022) [5]. From this dataset, we select 385 raw multibeam sonar images without augmentation and annotate them using LabelMe. The resolution of these images is 512 × 512. Some of the images and their corresponding annotations are shown in Figure 1. To enhance data complexity and improve model accuracy, we apply data augmentation after image partitioning. The augmentation operations include horizontal and vertical flipping, scaling, Gaussian noise addition, and random brightness adjustment. Among them, the scaling ratio range is 50–150%, the mean of Gaussian noise is 0, the standard deviation is 0.2, and the brightness adjustment factor range is 0.5–1.5. As a result, we obtain a total of 3080 images, which are then divided into a training set (2156 images), a validation set (616 images), and a test set (308 images).

### 3.2. YOLO-AR Model

YOLOv11 is the latest model released by Ultralytics. Compared to its predecessor, YOLOv8, its main innovations lie in the introduction of the C3k2 mechanism and the C2PSA module. The C3k2 module is an extension of C2f, functioning as a feature fusion module. C3k2 builds upon the C2f module by introducing a configurable parameter, C3k, which defines the module’s operational mode. When C3k is set to False, the module behaves like the original C2f with a conventional bottleneck design. When C3k is set to True, the bottleneck is replaced by the C3 module. This configurable design enhances the model’s adaptability, allowing it to better meet the varying demands of different detection tasks and application scenarios.

YOLOv11 introduces the C2PSA module—an enhanced version of the C2f module that integrates the Pointwise Spatial Attention (PSA) mechanism. This integration significantly boosts the model’s capacity to extract and emphasize critical features. The PSA mechanism uses a multi-head attention structure with a feedforward neural network, enabling the model to focus on important regions and filter out irrelevant information. The feedforward component further refines these features, enabling the network to capture more complex, nonlinear relationships. This type of attention mechanism proves especially effective in processing complex image data, as it helps the model concentrate on essential object characteristics, ultimately enhancing detection performance.

**Figure 2:**
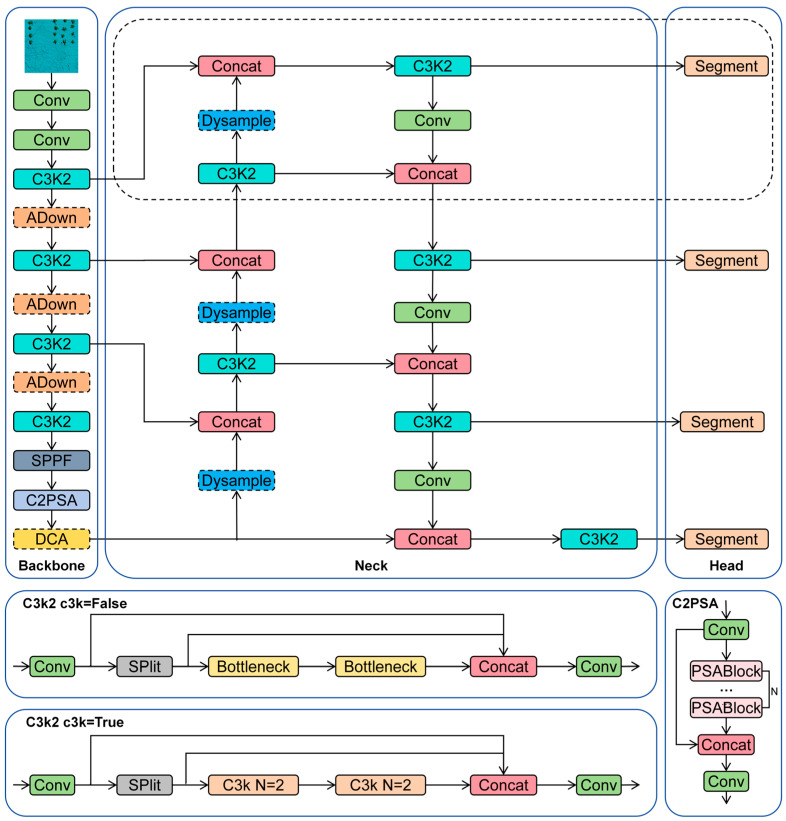
Structure diagram of YOLO-AR module.

In this study, YOLOv11 is chosen as the baseline model due to its high accuracy and low parameter count as the latest iteration of the YOLO series. However, when directly applied to artificial reef detection in complex seafloor environments, its performance is suboptimal. This is because multibeam sonar images differ significantly from natural images, exhibiting lower resolution, higher noise levels, fewer distinct target features, and varying artificial reef sizes—requiring both large and small object detection capabilities. To address these limitations and ensure precise detection, we modify the original YOLOv11 model specifically for artificial reef detection, naming the improved version YOLO-AR. As illustrated in Figure 2, the modified components are highlighted with dashed lines. To enhance the network’s focus on artificial reef features, we propose a spatial information attention mechanism module called DCCA and integrate it into the backbone network. Detailed information about the DCCA module can be found in Section 3.2.1. Additionally, to improve the model’s performance in detecting small artificial reefs in sonar images, we refine the neck structure and modify the upsampling module, with further details available in Section 3.2.2. Furthermore, to maintain model accuracy while reducing computational complexity, we replace the last two Conv layers in the backbone network with an innovative downsampling module (ADown). This modification enhances network precision while reducing the number of parameters. The specifics of the ADown module are detailed in Section 3.2.3.

#### 3.2.1. Improved Backbone Network with DCCA Module

Artificial reefs usually have specific geometric shapes. The Coordinate Attention mechanism can more accurately capture the edge and spatial distribution characteristics of the target through coordinate information. Coordinate Attention (CA) is an efficient attention module particularly suitable for lightweight network models. Based on traditional channel attention mechanisms (such as the SE module), the CA mechanism introduces spatial position information to generate direction-sensitive attention maps, thereby significantly improving the network’s capability in precise localization and feature representation. Unlike traditional attention mechanisms that compress features into a single vector via global pooling, CA employs two one-dimensional global pooling operations to encode features along the horizontal and vertical directions separately. This method not only captures long-range dependencies along one direction but also retains positional information in the other direction, which helps accurately characterize the spatial structure of target objects. The Coordinate Attention module encodes direction-aware information through two complementary attention maps—one emphasizing horizontal spatial relationships and the other focusing on vertical ones. These maps are applied to the input feature map via pixel-wise multiplication, allowing the network to better concentrate on regions of interest. Designed with efficiency in mind, the CA module is both lightweight and flexible, incurring minimal computational cost, which makes it well-suited for dense prediction tasks [37].

However, in complex underwater environments—particularly when dealing with the irregular shapes of artificial reefs and silt accumulation caused by dynamic water flows—Coordinate Attention (CA) can enhance the weighting of feature channels, but standard convolution remains limited in feature representation when sonar images exhibit high noise and weak textures. In contrast, dynamic convolution can adaptively adjust convolution kernels according to the input features, enabling better adaptation to diverse reef morphologies and noise patterns, thereby improving the model’s responsiveness to artificial reefs. Moreover, compared with deformable convolution (DCN), dynamic convolution incurs a lower computational overhead and does not require additional learning of sampling locations, making it more suitable for deployment in sonar-based tasks. To address this issue and enhance the segmentation performance, we propose an improved attention mechanism named DCCA (Dynamic Convolution Coordinate Attention). In DCCA, the standard convolution layers in CA are replaced with dynamic convolution operations [38]. Dynamic convolution is an emerging technique that adaptively generates convolution kernels based on individual input samples. This approach increases the network’s representational capacity without a proportional rise in computational cost. Unlike traditional static convolution, dynamic convolution utilizes a lightweight parameter generator to produce multiple weight coefficients from input features. These coefficients are subsequently used to dynamically combine a set of predefined convolution kernels. More specifically, each dynamic convolution operation begins by extracting global contextual information through average pooling, followed by a two-layer MLP that outputs kernel weight coefficients. These weights are normalized using a softmax function and utilized to construct the final dynamic convolution kernel. This mechanism captures richer feature relationships, enhances the model’s representation ability, and maintains a low computational cost. Moreover, dynamic convolution significantly improves model performance in small-sample scenarios, making it particularly effective for segmentation tasks involving complex seabed substrates of artificial reefs.

**Figure 3:**
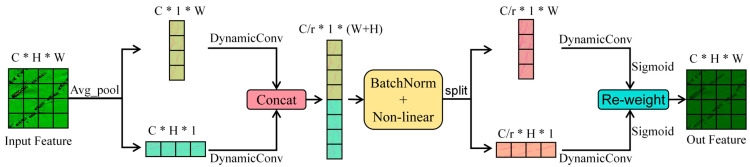
Structure diagram of DCCA module.

The structural diagram of the DCCA module is shown in Figure 3. The specific operation of the DCCA module involves performing two independent one-dimensional global pooling operations on the input feature map in the horizontal and vertical directions, aggregating them into two feature maps with specific directional and positional information, as shown in Equations (1) and (2):

(1) $$z_{c}^{h}\left(h\right)=\frac{1}{W}\sum_{0\leq i<W}x_{c}\left(h,i\right)$$

(2) $$z_{c}^{W}\left(h\right)=\frac{1}{H}\sum_{0\leq j<H}x_{c}\left(j,w\right)$$

The two feature maps are concatenated and passed through a convolution operation. The newly generated feature map is then batch normalized and activated non-linearly, producing a feature map *f* ∈ *R^C^*^/r*1*(*W*+*H*)^. Subsequently, *f* is split along the spatial dimension and processed using dynamic convolution. The outputs are then activated using the sigmoid function to obtain *g^h^* and *g^w^*, as shown in Equations (3) and (4):

(3) $$g^{h}=\sigma (F_{h}(f^{h}))$$

(4) $$g^{w}=\sigma (F_{w}(f^{w}))$$

where the *F_h_* and *F_w_* are the convolution operations and the *σ* is the sigmoid activation layer.

Finally, the obtained weights are element-wise multiplied with the original input image to generate the weighted attention feature map.

The DCCA model not only captures directional and positional information but also enhances sensitivity to diverse features through the high parameter capacity of dynamic convolution. This improvement translates into better segmentation accuracy and more precise target region identification in experiments, particularly in marine environments with complex textures and sparse targets.

#### 3.2.2. Small-Target Detection Neck with DySample Module

Due to the varied shapes of artificial reefs, some are relatively small in size. Additionally, as a result of sediment deposition caused by underwater currents, portions of artificial reefs may be buried by silt. The network faces challenges in effectively detecting these small artificial reefs. To enhance the detection of small targets in artificial reef segmentation, we improved the neck structure of the network. Traditional YOLO models perform predictions at different feature map scales; however, the large downsampling ratios limit the resolution for small targets, reducing detection accuracy. To address this issue, we designed a new branch in the network’s neck structure with a lower downsampling rate to preserve more detail information. Additionally, we introduced an extra detection head in the neck to better capture small target features.

Specifically, we incorporated an additional shallow feature map into the original network as input. After applying convolution operations for channel compression and feature fusion, a dedicated feature map for small-target detection was generated. This feature map integrates deep information with shallow spatial information, improving the model’s perception of small targets while maintaining computational efficiency. Finally, this branch, along with other scale-specific output layers, contributes to object detection, enhancing the model’s overall performance across multiple scales.

**Figure 4:**
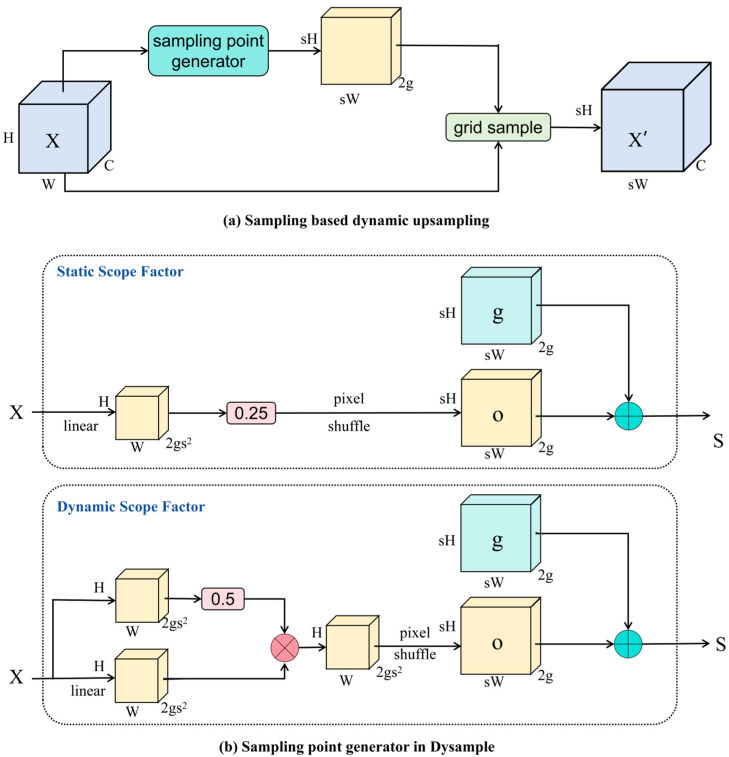
Sampling-based dynamic upsampling and sampling point generator designs in DySample.

Furthermore, we replaced the upsampling module in the neck structure with DySample [39], an efficient and lightweight dynamic upsampling module designed to address the high computational complexity and implementation challenges of existing dynamic upsampling methods. Its structure is illustrated in Figure 4, where Figure 4a represents the structure of DySample, based on dynamic sampling, where the sampling set is generated by a sampling point generator, and the input features are resampled by a grid sampling function. Figure 4b illustrates two types of sampling point generators: the static range factor version, in which offsets are generated by a linear layer, and the dynamic range factor version, in which a range factor is first generated and then used to modulate the offsets. Unlike FADE and SAPA, which rely on dynamic convolution kernels, DySample focuses on point sampling and resamples the feature map by generating content-aware offsets. This design avoids the high computational cost of dynamic convolution, eliminates the need for high-resolution guidance features, and is implemented using PyTorch’s built-in functions without requiring additional CUDA optimizations. As a result, DySample significantly reduces the model’s parameter count, computation cost, and memory usage. DySample achieves a computational efficiency comparable to traditional bilinear interpolation while delivering superior performance improvements in various dense prediction tasks. In artificial reef segmentation, conventional upsampling modules may introduce blurring or pseudo-textures in sonar noise scenarios, whereas DySample dynamically generates sampling weights based on local features and adaptively adjusts sampling points. This allows it to effectively capture the complex boundaries and textural details of reefs in challenging underwater environments. Its dynamic sampling mechanism ensures that the upsampled feature maps are more accurately aligned with the true boundaries, thereby improving segmentation accuracy and detail preservation. Moreover, unlike multi-scale feature fusion refinements, DySample does not introduce additional feature branches but directly enhances the quality of upsampling, reducing redundant computation. Its lightweight design is particularly well-suited for processing large-scale, high-resolution underwater data, in improving computational efficiency while lowering hardware resource requirements.

#### 3.2.3. Reduce Network Parameters with ADown Module

Sonar images have a high resolution. Directly using deep trunks to extract features would introduce a large amount of redundant computation. In YOLOv9, there is an ADown (Advanced Downsampling) module [40]. In a novel development, it combines average pooling and max pooling in a smart way. This helps to extract and keep valuable feature information more effectively. We achieve network lightweighting by replacing the downsampling module in the network backbone with ADown, and thereby reducing model complexity while retaining key information.

**Figure 5:**
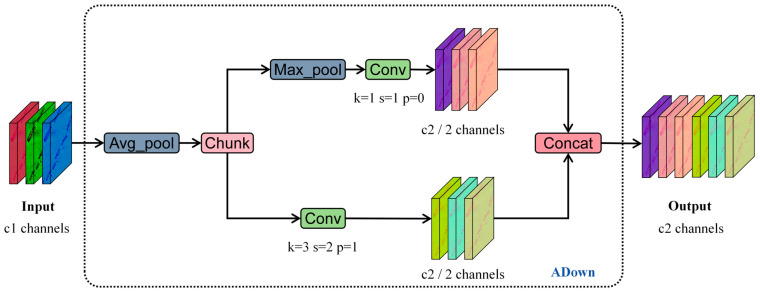
Structure diagram of ADown module.

As shown in Figure 5, the ADown module starts by using average pooling on the input feature map. After that, the feature map is split equally along the channel dimension. One half goes through a 3 × 3 convolution for downsampling. The other half is first processed by max pooling and then goes through a 1 × 1 convolution. This dual-branch design allows the model to use different types of feature cues at the same time. Average pooling can produce a smoothed and global representation. On the other hand, max pooling highlights sharp and prominent features. The outputs from these two branches are finally combined. This results in a feature map that has a lot of details and structural diversity.

Compared to traditional downsampling methods, ADown has several important advantages. Its design can not only make it more flexible to keep the spatial and channel-level information but also maintain computational efficiency. The parallel-branch structure of the module allows it to reduce the resolution. At the same time, it can keep the important characteristics of features. This makes it very suitable for applications like object detection and image segmentation. In these applications, it is crucial to preserve detailed features. In actual application, ADown has shown really great effectiveness. Standard downsampling operations often lead to information loss. It addresses this common problem. By doing so, it can provide more representative feature maps to the downstream network layers. As a result, it can improve the model’s performance in complex visual tasks.

## 4. Results

### 4.1. Experimental Hardware Configuration and Parameter Setting

The hardware and software configurations, along with experimental parameters, are detailed in Table 1. The experiments were conducted on a system equipped with an Intel Core i7-9750H manufactured by Intel Corporation (Santa Clara, CA, USA) and an NVIDIA RTX 4090D GPU (24 GB memory) manufactured by NVIDIA Corporation (Santa Clara, CA, USA). The software environment included Python 3.8, PyTorch 2.0.0, and CUDA 11.8. For training, the following parameter settings were adopted: an initial learning rate of 0.01, a batch size of 16, 200 training epochs, a momentum value of 0.937, and a weight decay of 0.0005. To ensure the consistency and reliability of the results, all experiments were conducted under the same hardware and parameter settings throughout the research process, and all models in this study were trained from scratch.

### 4.2. Precision Evaluation Index

In deep learning-based segmentation tasks, commonly used accuracy evaluation metrics include precision (P), recall (R), mean average precision (mAP), F1 score, and intersection over union (IOU).

Precision refers to the proportion of instances predicted as positive samples that are actually positive. A high precision score indicates that the model is more accurate in predicting positive samples. The calculation formula is as follows:

(5) $$P=\frac{TP}{TP+FP}$$

Recall refers to the proportion of actual positive samples that the model correctly identifies as positive. A high recall score indicates that the model can detect most positive samples with a low false negative rate. The calculation formula is as follows:

(6) $$R=\frac{TP}{TP+FN}$$

where TP (True Positive) represents the number of correctly predicted positive samples, FP (False Positive) represents the number of incorrectly predicted positive samples, and FN (False Negative) represents the number of actual positive samples that were incorrectly predicted as negative.

The mean average precision (mAP) is used to measure the model’s detection accuracy across different thresholds. mAP is the average of the average precision (AP) values across all categories. Since this study involves a single class of reef targets, mAP is equal to AP. AP is computed as the area under the precision–recall (P-R) curve using integration, and its formula is as follows:

(7) $$AP=\int_{0}^{1}P\left(R\right)dR$$

The F1 score combines precision and recall and is their harmonic mean. The F1 score is calculated as follows:

(8) $$F1=\frac{2PR}{P+R}$$

Intersection over union (IOU) is used to measure the overlap between the model’s predicted region and the ground-truth region. The IOU calculation formula is as follows:

(9) $$IOU=\frac{TP}{TP+FP+FN}$$

### 4.3. Analysis of Artificial Reef Segmentation Results

**Figure 6:**
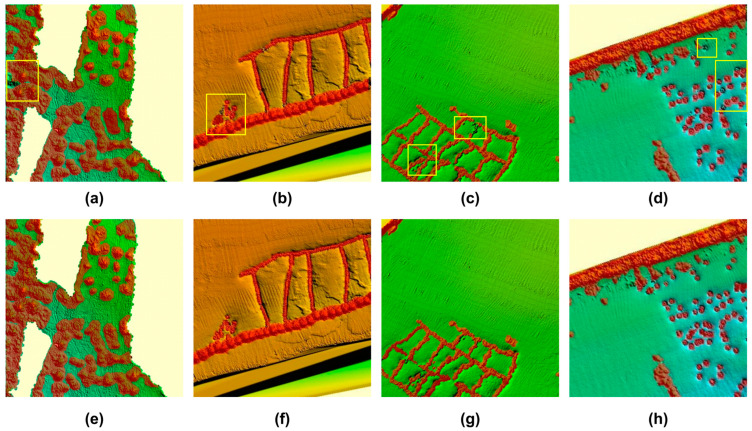
The segmentation results of an artificial reef using YOLOv11 (**a**–**d**) and YOLO-AR (**e**–**h**). The red area represents the artificial reefs segmented by the model.

Accurately detecting artificial reefs in multibeam sonar images is crucial for reef deployment and management. To evaluate the detection performance of YOLO-AR, artificial reefs were detected in the test set of multibeam sonar images. Figure 6 presents the detection results of artificial reefs using YOLOv11 and YOLO-AR. As shown in Figure 6, the yellow boxes indicate missed detections by YOLOv11, while YOLO-AR accurately detects these reefs. YOLO-AR effectively segments artificial reefs in multibeam sonar images, demonstrating a superior performance over YOLOv11. Furthermore, YOLO-AR can be applied to detect most continuous artificial reef groups.

To further quantitatively evaluate the artificial reef detection capability of YOLO-AR, Table 2 presents the detection results of YOLOv11 and YOLO-AR. As shown in Table 2, the precision, recall, mAP@0.5, mAP@0.5-0.95, IOU, and F1 score of YOLO-AR reached 0.939, 0.879, 0.912, 0.601, 0.832, and 0.908, respectively, which are 0.046, 0.085, 0.059, 0.048, 0.107, and 0.07 higher than those of YOLOv11. Additionally, the parameter count was reduced by 162,259. These results indicate that, compared to YOLOv11, the YOLO-AR model not only achieves a better artificial reef detection performance with higher accuracy but also requires fewer parameters.

### 4.4. Visual Evaluation of YOLO-AR by Grad-CAM

**Figure 7:**
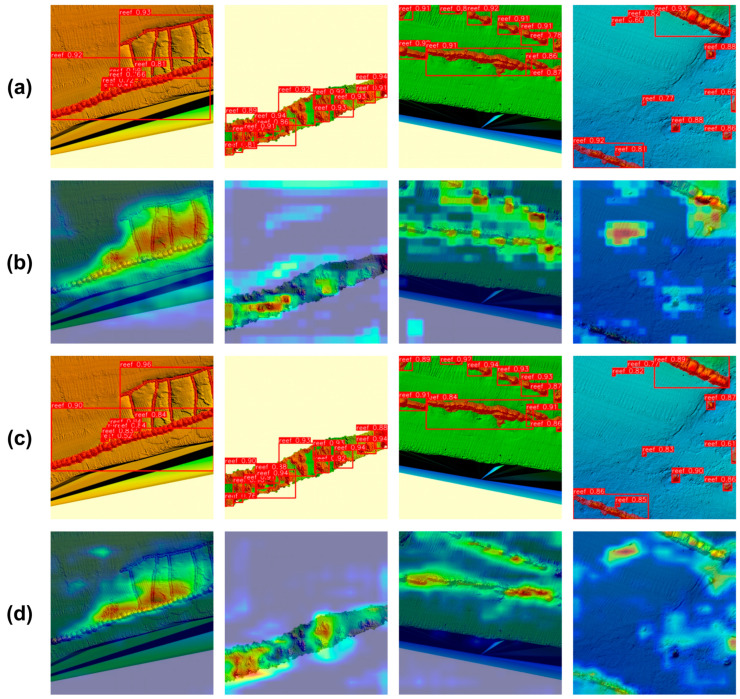
Visualization of artificial reef segmentation heatmaps of YOLOv11 and YOLO-AR. (**a**) Segmentation results of YOLOv11, (**b**) segmentation heatmap of YOLOv11, (**c**) segmentation results of YOLO-AR, and (**d**) segmentation heatmap of YOLO-AR.

**Figure 8:**
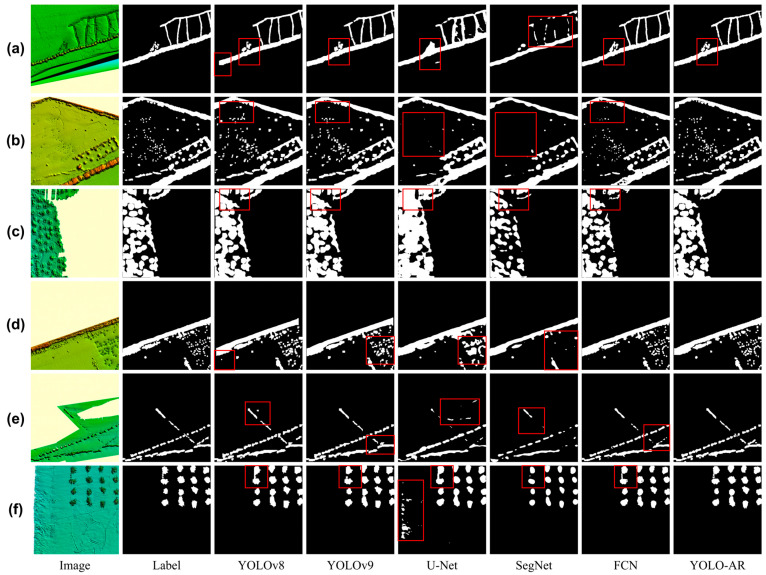
Comparison of multibeam sonar image artificial reef segmentation results among six segmentation models. (**a**–**f**) are six images in the test set. The first two columns are sonar images and real labels respectively. The following six columns show the artificial reef segmentation results of YOLOv8, YOLOv9, U-Net, SegNet, FCN and YOLO-AR. The red box represents the key comparison areas of the segmentation results.

To fully evaluate the performance of YOLO-AR in detecting artificial reefs in multibeam sonar images, we visualized the heatmaps generated by Gradient-weighted Class Activation Mapping (Grad-CAM) for YOLO-AR and YOLOv11, as shown in Figure 7. These heatmaps provide a visualization of artificial reef detection results. All sonar images in Figure 8 are sourced from the test set of the artificial reef detection dataset. Warm colors in the heatmaps highlight regions with a greater contribution to artificial reef detection.

As shown in Figure 7a,b, when using YOLOv11 to segment artificial reefs, the model is highly sensitive to background interference, leading to reduced attention to the reef structures. This is a major reason why small artificial reefs are often missed or misclassified when using YOLOv11 for detection. However, after optimization, the heatmaps of YOLO-AR reveal a significant enhancement in attention to artificial reef regions while suppressing irrelevant background information (see Figure 7c,d). This observation confirms the effectiveness of the optimizations, demonstrating that YOLO-AR can strengthen its focus on artificial reefs while mitigating background interference. These optimizations prove that the model can effectively utilize contextual information, enhancing attention to artificial reefs while reducing distractions from irrelevant background regions. The visualization results from Grad-CAM confirm that these improvements contribute to a better artificial reef detection performance in multibeam sonar images.

### 4.5. Model Parameter Evaluation

To evaluate the model size, Table 3 presents the parameter count, model size, and FLOPs of six mainstream segmentation models (YOLOv8, YOLOv9, U-Net, SegNet, FCN, and YOLO-AR). FLOPs (floating point operations) represent the number of computations required by an algorithm and serve as a measure of its complexity. The parameter counts of the six segmentation models are 3.26 million, 27.84 million, 24.59 million, 29.46 million, 18.64 million, and 2.67 million, with YOLO-AR having the fewest parameters among them. The model sizes of the six segmentation models are 6.46 MB, 106.91 MB, 94.97 MB, 337.45 MB, 269.74 MB, and 5.58 MB, with YOLO-AR being the smallest. The FLOPs for the six models are 12.1 G, 159.1 G, 361.85 G, 327.13 G, 203.99 G, and 23.2 G, where YOLO-AR has slightly higher FLOPs than YOLOv8 but significantly lower than the other four models. The results demonstrate that YOLO-AR has the fewest parameters and the smallest model size. Although its FLOPs are slightly higher than YOLOv8, it remains the most lightweight among the six segmentation models overall.

### 4.6. Performance Comparison Experiment of the Mainstream Segmentation Model

To further evaluate the performance of YOLO-AR, we compared it with mainstream segmentation models, including FCN, U-Net, YOLOv12, SegNet, and YOLOv8. Table 4 presents the evaluation metric comparisons of these six segmentation models. The mAP@0.5 values for the six segmentation models are 0.842, 0.851, 0.820, 0.822, 0.718, and 0.912, respectively. YOLO-AR achieves the highest mAP among all the models, indicating its superior ability to accurately segment the target. The IOU values for the six segmentation models are 0.714, 0.733, 0.747, 0.798, 0.683, and 0.832, respectively. YOLO-AR has the highest IOU value among the six models, demonstrating the highest spatial overlap between predictions and ground truth, with the most precise boundary alignment. The F1 scores for the six segmentation models are 0.833, 0.846, 0.855, 0.888, 0.812, and 0.908, respectively. YOLO-AR achieves the highest F1 score, indicating the best balance between segmentation confidence and minimizing missed detections. The experimental results show that among the six segmentation models, YOLO-AR has the best performance, making it the most suitable for artificial reef segmentation in multi-beam sonar images.

## 5. Discussion

### 5.1. Ablation Experiment

The ablation experiment was conducted by selectively disabling the DCCA module, ADown module, and the improved neck structure (denoted as DNeck) in YOLO-AR to observe their impact on performance. This approach aimed to validate the necessity of these feature enhancements. Additionally, to ensure the accuracy of the ablation experiments, all tests were performed under the same environment and with identical hyperparameters. The results of the ablation study for the YOLO-AR model are presented in Table 5. Notably, a ✓ symbol indicates an enabled module, while a × symbol represents a disabled module.

From the results in Table 5, it is evident that the DCCA module, ADown module, and the improved neck structure all provided positive contributions to artificial reef detection. Specifically, after incorporating the DCCA module into the model, the recall, mAP@0.5, mAP@[0.5:0.95], and IOU increased by 0.012, 0.008, 0.012, and 0.009, respectively. When improving the neck structure of YOLOv11, the precision, recall, mAP@0.5, mAP@0.5-0.95, IOU, and F1 scores increased by 0.032, 0.069, 0.046, 0.011, 0.076, and 0.052, respectively. However, the introduction of DCCA and the improvement in neck structure have led to an increase of 0.1 million and 0.08 million parameters, respectively.

To address this, the ADown module was introduced into the network. Although this slightly reduced some evaluation metrics, it significantly reduced the model’s parameter count by 0.34 million. After integrating ADown into the network and improving the neck structure, the precision, recall, mAP@0.5, mAP@[0.5:0.95], IOU, and F1 scores reached 0.930, 0.866, 0.902, 0.581, 0.813, and 0.897, respectively, outperforming the configuration with only the improved neck structure. This improvement is likely because the ADown module, by reducing feature map dimensions and working with the small-object detection layer, was able to extract more discriminative features. Meanwhile, DySample facilitated a more effective recovery of high-resolution details during the upsampling process. This combination likely helped strike a better balance between high-level features and low-level details, thereby improving the overall model accuracy. Without ADown, feature complexity and redundancy could increase, leading to performance degradation. However, incorporating ADown helped compress the network and extract more useful features, synergizing well with the optimized neck structure to achieve a superior performance.

For the final YOLO-AR model, which integrates the DCCA module, ADown module, and the improved neck structure, the precision, recall, mAP@0.5, mAP@[0.5:0.95], IOU, and F1 scores reached 0.939, 0.879, 0.912, 0.601, 0.832, and 0.908, respectively, achieving the best performance among all tested models. Additionally, this configuration maintained a relatively small parameter count of 2.67 million while achieving the highest accuracy. The ablation study results confirm the effectiveness of the DCCA module, ADown module, and DNeck structure in the YOLO-AR model.

### 5.2. Visual Evaluation of Artificial Reef Segmentation by Different Models

To evaluate the artificial reef segmentation performance of YOLO-AR on real sonar images, a visual comparison was conducted between YOLO-AR and five other segmentation models using test set images. As shown in Figure 8, we plotted a binary segmentation graph based on the segmentation results of the six models to better observe the segmentation effects of each model. In Figure 8, the first column shows the sonar images in the test set, and the second column presents the true labels of the artificial reefs. The following six columns display the artificial reef segmentation results of YOLO-AR, YOLOv9, YOLOv8, U-Net, SegNet, and FCN. The red rectangles in the figure indicate regions where the models exhibit a poor segmentation performance for artificial reefs.

In Figure 8a, YOLOv8 fails to detect artificial reefs located at the edges of the image. SegNet produces incomplete detections for continuous reefs in the image. YOLOv9, U-Net, and FCN struggle with detecting smaller reef targets. Although YOLO-AR also exhibits some deficiencies in reef segmentation within the red rectangles, it performs the best among the six segmentation models. In Figure 8b, YOLOv8, YOLOv9, and FCN incorrectly classify background areas as artificial reefs. U-Net and SegNet fail to detect large portions of small reef targets. YOLO-AR accurately detects large clusters of small reef targets in the image. In Figure 8c, YOLOv8, YOLOv9, and U-Net misclassify the background within the red rectangle as reefs. U-Net struggles with segmenting closely spaced reefs, resulting in merging errors. SegNet and FCN suffer from missed detections. YOLO-AR, on the other hand, provides segmentation results that closely match the actual reef boundaries. In Figure 8d, YOLOv8 produces incorrect detections, while YOLOv9 and U-Net fail to effectively segment closely spaced reefs. SegNet misses large portions of small reef targets. YOLO-AR successfully segments both small reefs and closely spaced reefs with high accuracy. In Figure 8e, YOLOv8 and U-Net mistakenly classify background areas as reefs, while YOLOv9, SegNet, and FCN suffer from missed detections. YOLO-AR’s segmentation results closely align with the ground truth labels. In Figure 8f, YOLOv8, U-Net, and FCN struggle to segment closely spaced reefs, with U-Net also misclassifying parts of the background as artificial reefs. YOLOv9 and SegNet exhibit missed detections. YOLO-AR does not suffer from these issues and delivers an excellent segmentation performance.

The visual evaluation results demonstrate that YOLO-AR outperforms the other five segmentation models in segmentation of artificial reefs in multibeam sonar images. YOLO-AR accurately detects both large and small reef targets and effectively segments the boundaries of closely spaced reefs.

### 5.3. Research Limitations and Future Prospects

The proposed model achieves efficient and accurate segmentation of artificial reefs in multibeam sonar images. However, this study still has certain limitations. First, artificial reefs exist in various shapes, such as cubes and triangular pyramids, but the dataset used in this work contains only a single reef type, meaning it is currently unable to differentiate multiple categories of reefs. Second, due to hydrodynamic erosion, artificial reefs may gradually become buried over time, leading to variations in reef sizes within images, which poses a challenge for accurate detection. Although a small-object detection layer was introduced to address this issue, its specific effectiveness remains unclear. In the future, we plan to conduct in situ surveys in marine ranches to collect more diverse artificial reef data, enabling the model to distinguish reefs of different shapes and conditions. Additionally, during data annotation, reefs of different sizes will be labeled separately to better investigate the impact of various improvement strategies. Furthermore, by incorporating quantitative indicators such as the number or area of detected reefs, the model could be extended to estimate the utilization rate of artificial reef areas, thereby contributing to more effective planning and maintenance of artificial reef structures.

## 6. Conclusions

Accurate detection of artificial reefs in sonar images using deep learning models is of great significance for artificial reef monitoring and maintenance. In this study, we propose a deep learning model, YOLO-AR, capable of precise and efficient edge detection of artificial reefs. Built upon YOLOv11, YOLO-AR incorporates several key improvements. First, we introduce the attention module DCCA to enhance the model’s focus on artificial reef regions. Second, we modify the network’s neck structure by adding a small-object detection layer and integrating the dynamic upsampling module DySample, improving the model’s ability to detect smaller reefs. Finally, to reduce the parameter count, we replace some convolutional layers in the backbone with the lightweight ADown downsampling module. Experimental results demonstrate that YOLO-AR achieves a precision of 0.939, a recall of 0.879, an mAP@0.5 of 0.912, an mAP@[0.5:0.95] of 0.601, an IOU of 0.832, and an F1 score of 0.908, with a parameter count of only 2.67 million. Comparisons with mainstream deep learning models show that YOLO-AR achieves a high level of accuracy while maintaining the smallest model size and the lowest parameter count, effectively balancing detection accuracy and model complexity. It accurately detects artificial reefs of different sizes, whether they are continuous or isolated. In future work, we plan to expand the dataset and further optimize YOLO-AR to adapt the model for detecting and distinguishing various types of artificial reefs.

## Figures and Tables

**Table 1:** Experimental software and hardware configuration and training parameters.

Hardware/Software	Configuration	Training Parameter	Configuration
CPU	Intel Core i7-9750H	Initial learning rate	0.01
GPU	NVIDIA RTX4090D	Momentum	0.937
Python	3.8.10	Weight decay	0.0005
Pytorch	2.0.0	Bach size	16
Cuda	11.8	Learning epoch	200

**Table 2:** Performance comparison of artificial reef segmentation between YOLOv11 and YOLO-AR.

Model	P	R	mAP@0.5	mAP@[0.5:0.95]	IOU	F1	Parameters
YOLOv11	0.893	0.794	0.853	0.553	0.725	0.841	2834763
YOLO-AR	0.939	0.879	0.912	0.601	0.832	0.908	2672504

**Table 3:** The parameter, size, and FLOPs of six segmentation models. The bold numbers represent the minimum value in each column.

Model	Parameters (Million)	Model Size (MB)	FLOPs (G)
YOLOv8	3.26	6.46	**12.1**
YOLOv9 [40]	27.84	106.91	159.1
U-Net [41]	24.89	94.97	361.85
SegNet [42]	29.46	337.45	327.13
FCN [43]	18.64	269.74	203.99
YOLO-AR	**2.67**	**5.58**	23.2

**Table 4:** Performance comparisons of the six segmentation models. The bold numbers represent the maximum value in each column.

Model	P	R	mAP@0.5	IOU	F1
YOLOv8	0.887	0.786	0.842	0.714	0.833
YOLOv9 [40]	0.893	0.804	0.851	0.733	0.846
U-Net [41]	0.873	0.838	0.820	0.747	0.855
FCN [42]	0.966	0.821	0.822	0.798	0.888
SegNet [43]	**0.941**	0.714	0.718	0.683	0.812
YOLO-AR	0.939	**0.879**	**0.912**	**0.832**	**0.9** **08**

**Table 5:** Ablation experiment results of different modules.

YOLOv11	DCCA	ADown	DNeck	P	R	mAP@0.5	mAP@[0.5:0.95]	IOU	F1	Parameters (Million)
✓	×	×	×	0.893	0.794	0.853	0.553	0.725	0.841	2.83
✓	✓	×	×	0.892	0.806	0.861	0.565	0.734	0.847	2.93
✓	×	✓	×	0.891	0.788	0.848	0.539	0.719	0.836	2.49
✓	×	×	✓	0.925	0.863	0.899	0.564	0.801	0.893	2.91
✓	✓	✓	×	0.891	0.801	0.856	0.564	0.730	0.844	2.57
✓	✓	×	✓	0.923	0.866	0.902	0.574	0.808	0.894	2.99
✓	×	✓	✓	0.930	0.866	0.902	0.581	0.813	0.897	2.57
✓	✓	✓	✓	0.939	0.879	0.912	0.601	0.832	0.908	2.67

## Data Availability

The dataset of this article and the FIO-AR dataset can be downloaded at https://pan.baidu.com/s/1nCqWAKxWE6kC4pAPzfPtFw (password: abcd) (accessed on 30 July 2025). The source code of YOLO-AR can be obtained from the corresponding author.
